# Spatiotemporal disparities in automated external defibrillator access: identifying national deficits

**DOI:** 10.1016/j.resplu.2025.101135

**Published:** 2025-10-15

**Authors:** Sarah Maria Esther Jerjen, Armin Gemperli

**Affiliations:** University of Luzern, Faculty of Health Sciences and Medicine, Alpenquai 4, 6005 Luzern, Switzerland

**Keywords:** Automated External Defibrillator (AED), Geographic Information Systems (GIS), Bayesian spatial modeling, Accessibility, Clustering, Employment, Population

## Abstract

**Background:**

Timely defibrillation is vital for survival after out-of-hospital cardiac arrest (OHCA), yet the availability of automated external defibrillators (AEDs) at critical moments remains uncertain. Placement guidelines emphasize high-traffic public sites, but most OHCAs occur at home and outside business hours, raising questions about accessibility. Few national studies address both geography and temporal availability. Switzerland’s decentralized system without placement standards provides a critical test case. This study applies a spatial–statistical framework to analyze clustering, assess links between AED presence and population or employment density, and quantify accessibility deficits and affected populations.

**Methods:**

We analyzed over 14,000 devices from the Swiss AED registry under two scenarios: those with 24-h access and all devices hypothetically available 24/7. Clustering was tested with Ripley’s L and Moran’s I. Associations with population or employment density were estimated using Bayesian spatial logistic models (BYM2), separating demographic effects from residual spatial structure. Accessibility deficits were defined as the gap between modeled and observed AED presence, weighted by demographic counts, and expressed as population-weighted risk scores.

**Results:**

AEDs were significantly spatially clustered, with deployment more strongly aligned with employment than with residential population. Strong residual spatial structuring persisted beyond demographic effects. The 5 % of grid cells with the highest accessibility deficits contained between 16 % and 42 % of the national population or workforce.

**Conclusion:**

AED deployment in Switzerland reflects historical and institutional patterns rather than risk-based planning. This study provides a framework for identifying spatial and temporal accessibility deficits and guiding more equitable AED placement.

## Highlights

• National-scale Bayesian spatial analysis of AED deployment patterns.
• AEDs correlate more with workplaces than homes despite 68 % of arrests at home.
• Quantification of spatial variation resulting from non-evidence-based AED placement.
• Top 5 % underserved areas contain 42 % of population, revealing systematic inequities.
• Temporal access restrictions amplify geographic disparities in AED availability.

## Introduction

Early defibrillation, through timely access to automated external defibrillators (AEDs), is one of the most effective interventions for survival following out-of-hospital cardiac arrest (OHCA).^1^, ^2^ Survival declines sharply with each minute of delay, and AED use by bystanders can double survival rates.^3^, ^4^ Yet, the potential impact of AEDs is constrained by unequal access: OHCA survival remains below 10 % in most settings, with persistent spatial disparities.^5^, ^6^ Residential and rural areas often face longer retrieval times, fewer devices, or restricted access during evenings and weekends, reducing timely defibrillation.^7^, ^8^

International studies confirm that AED accessibility directly affects survival. In Denmark, arrests near an accessible AED were nearly three times more likely to receive bystander defibrillation and had almost double the 30-day survival compared with inaccessible devices.^9^ In Stockholm, one-month survival reached 70 % when a public AED delivered the first shock, compared with 31–42 % when defibrillation was initiated only by EMS.^10^ More broadly every minute of delay to defibrillation is associated with an ∼9 % decrease in neurologically intact survival, underscoring why access and time-to shock are central to outcome.^11^

These findings have directly informed international strategies, as promoted by the European Resuscitation Council and the Red Cross, which have prioritized AED placement in high-visibility, high-traffic sites.^12^, ^13^, ^14^, ^15^ While this has improved AED availability in public spaces, it has not addressed the lack of access at home and outside business hours where most OHCAs occur.^16^, ^17^ Many AEDs are locked inside business buildings and are inaccessible to the public, raising the question of whether AED infrastructure reflects actual needs. The national-scale implications of this divergence, including the spatial and temporal dimensions, remain understudied.

Switzerland provides a critical setting to examine AED accessibility because deployment is decentralized, relying on communal authorities, foundations and private actors, and must serve a heterogeneous geography spanning dense urban centers, rural areas, and mountain regions. Despite comparatively large numbers of AEDs and a national goal of achieving AED use in 50 % of OHCAs, bystander defibrillation rates remain at about 10 %.^16^, ^18^ The absence of a national mandate for spatial planning or coordinated oversight has led to fragmented deployment.^19^ Many devices lack 24-h access, limiting usefulness in residential and off-hour settings.^20^ Evaluating placement against demand and access times is essential since even well-sited devices are useless if inaccessible at the moment of arrest. Although focused on Switzerland, the approach applied here is transferable to other decentralized health systems facing similar challenges in AED deployment.

This study uses a national AED registry and demographic data to evaluate whether AED deployment aligns with demographic need. Specifically, it aims to:

1. Assess whether AED placement follows Complete Spatial Randomness (CSR, i.e., random scatter) or exhibits clustering patterns;
2. Evaluate associations between AED presence and population or employment density to determine alignment with residential areas and economic hubs;
3. Develop a spatial risk model to quantify AED accessibility deficits and identify high-need areas for optimized deployment; and
4. Produce targeted maps to support the systematic prioritization of regions for future AED placement interventions.

Through this analysis, the study provides empirical evidence on the spatial structure of AED availability and accessibility and offers a framework for guiding future deployment toward more risk-aligned, population-responsive strategies.

## Methodology

### Study design and data processing

AED data were obtained from *Defikarte.ch* (accessed December 2024), comprising 5,667 AEDs accessible 24-h per day (39 % of devices) and 8,745 AEDs with restricted hours (61 % of devices).^20^ Analyses were performed for the 24-h available AEDs (referred to as: 24-h-AEDs) and a hypothetical scenario in which all AEDs, including those with restricted hours, were assumed to be accessible 24-h (referred to as: all-AEDs). The analysis used a standardized 1 km^2^ grid covering Switzerland, with all spatial data projected to the Swiss coordinate system (EPSG:2056). This resolution ensured consistency across datasets and aligns with the Eurostat GEOSTAT standard, which represents census data in a 1 km^2^ grid across Europe.^21^

Demographic information was derived from vector-based settlement data provided by the Federal Office for Spatial Development. The settlement data were aggregated to the 1 km^2^ grid and spatially intersected to identify built-up grid cells.^22^ Hectare-level population data (100 m^2^ resolution) from the Federal Statistical Office (FSO, 2023) were summed to calculate population within each 1 km^2^ cell. Similarly, rasterized employment data from the 2022 FSO Business Census (also at 100 m^2^ resolution) were aggregated to represent full time equivalent jobs per grid cell. Spatial processing and statistical modeling were performed using QGIS 3.32 and R 4.3.2, with sf, spdep, INLA, terra, exactextractr, and tmap packages.

### Complete spatial randomness assessment

To determine whether AEDs were randomly distributed within settled areas, spatial clustering was evaluated using Ripley’s L-function, using 99 Monte Carlo simulations with isotropic edge correction, across scales from 0 to 3000 m. CSR was treated only as a descriptive baseline: while clustering is expected in built environments, deviation from CSR motivates subsequent spatial modeling. If the distribution statistically significantly deviated from CSR, global spatial autocorrelation was assessed using Moran’s I, calculated from AED density values aggregated to the 1 km2 grid. To mitigate edge effects, the grid was extended 1 km beyond the AED bounding box. A spatial weights matrix on first-order Queen contiguity defined neighborhood structure. Statistically significantly positive Moran’s I (*p* < 0.05) indicated clustering, values near zero spatial randomness. In plain terms, Ripley’s L tests whether AEDs are more clumped than random, and Moran’s I checks whether neighboring grid cells tend to look alike.

### Bayesian spatial modeling

To examine the association between AED presence and population or employment density, a Bayesian hierarchical logistic model was implemented using the Besag-York-Mollié (BYM2) framework estimated via Integrated Nested Laplace Approximation (INLA) and empirical Bayes mode selection.^23^ For each 1 km^2^ grid cell, AED presence was modeled as a binary outcome, with population or employment density as the predictor, standardized for convergence and effect comparability. This framework was selected because it distinguishes random noise from systematic geographic clustering, providing more robust inference than simpler regression approaches.

The model was specified as:

$$\text{logit}\left(P\left(Y_{i}=1\right)\right)=\beta_{0}+\beta_{1}X_{i}+\theta_{i},\;\;with$$

$$\theta_{i}=\sqrt{\frac{\Phi }{\tau }}u_{i}+\sqrt{\frac{1-\Phi }{\tau }}v_{i}$$

where

• i=1,2,3,⋯ are the 1 km^2^ grid cells,
• $Y_{i}$ and $X_{i}$ are indicators of presence of at least one AED at grid cell i, and standardized population or employment density, respectively,
• $u_{i}∼\;ICAR$ captures structured spatial autocorrelation using Queen contiguity,
• $v_{i}\;∼N\left(0,\sigma^{2}\right)$ represents spatially unstructured heterogeneity,
• $\Phi \in \left[0,1\right]$ is a mixing parameter indicating how much of the unexplained variation follows spatial structure,
• τ = overall precision. The spatially structured ($u_{i}$) and unstructured ($v_{i}$) random components were standardized to variance one, ensuring that τ represents the overall precision of the spatial random effect.

After model fitting, the posterior distribution of the spatial mixing parameter Φ was extracted for each model to quantify the degree of spatially structured residual variation. Posterior means and 95 % credible intervals (CrI) were reported using equal-tailed quantiles (2.5 % and 97.5 %).

Separate models were run for four scenarios:

1. 24-h-AEDs and population density as predictor
2. All-AEDs and population density as predictor
3. 24-h-AEDs and employment density as predictor
4. All-AEDs and employment density as predictor

In addition to the four predictor-based BYM2 models, two intercept-only models (one per AED dataset) were fitted to isolate and quantify latent spatial structure in the absence of demographic predictors. Unlike Ripley’s L or Moran’s I, which describe clustering, these models quantify the share of residual variation that follows a structured spatial pattern.

### AED deficit and risk scoring

To identify spatial disparities in AED coverage, a risk model was developed by computing the difference between predicted probabilities of AED presence, derived from BYM2 models and the observed binary presence indicators (0 or 1). For each 1 km^2^ grid cell, this difference represents an accessibility deficit ranging from −1 to +1. Positive values indicate under-coverage (AED expected but absent), and negative values indicating over-coverage (AED present despite low modeled expectation). This measure captures spatially contextualized accessibility deficits, allowing direct interpretation of unmet need in probabilistic terms.

Accessibility deficits were multiplied by population or employment count to produce demographically weighted risk scores, expressed in persons per km^2^. These scores quantify how many people or workers in a given area are underserved based on a mismatch between expected and actual AED presence. High positive scores in cells without an AED indicate many individuals living or working in a location where an AED would be expected but is missing. While not direct measures of physical inaccessibility, they estimate the number of people likely affected by inadequate AED deployment, given the local demographic demand.

This procedure was applied across all four model scenarios (24-h vs. all-AEDs; population vs. employment predictors). High-risk areas were defined as the top 5 % of grid cells with the highest demographically weighted risk scores, representing the largest underserved populations or workforces. These cells were summarized descriptively to quantify the share of the national population or workforce affected by the most acute deployment shortfalls under each scenario.

Spatial spillover was evaluated by testing autocorrelation of demographically weighted risk scores. Using a first-order Queen contiguity matrix, each cell’s score was compared to the mean of its neighboring scores. Pearson correlation coefficient quantified clustering with positive values indicating that high risk-areas tend to occur in geographic clusters. All analyses were constrained to Swiss national boundaries, and results visualized using interactive choropleth maps to support geographically targeted AED deployment strategies.

## Results

### Spatial distribution of AEDs

**Fig. 1:**
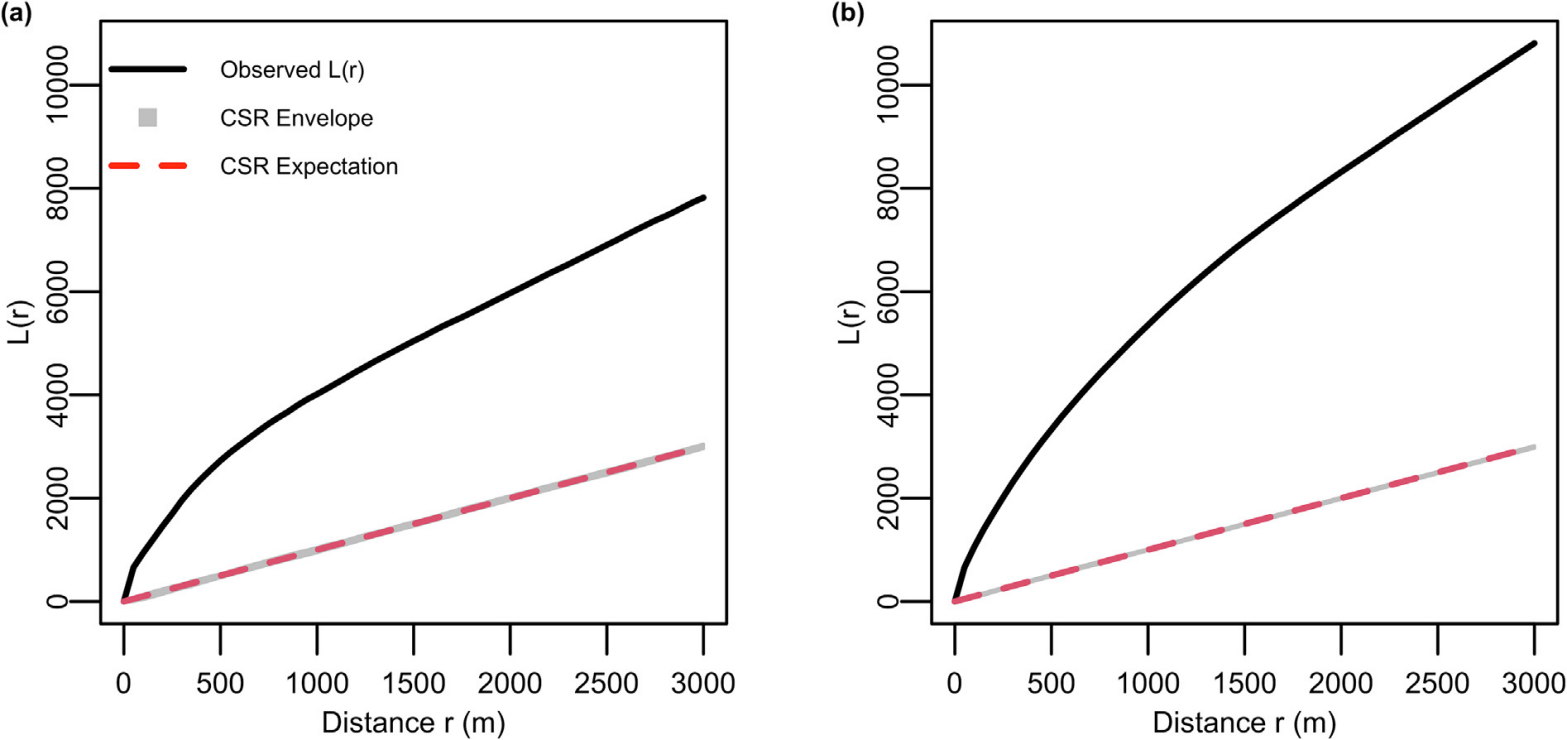
Ripley’s L-functions within settled areas; (a) 24-h-AEDs. (b) All-AEDs. The *x*-axis indicates spatial scale, representing the radius of analysis in meters. The *y*-axis shows the deviation of observed AED clustering from expectations under complete spatial randomness (CSR), measured by *L*(*r*). Solid black lines show the observed L-values, while gray bands represent the 99 % simulation envelopes under CSR. The red dashed line marks the expected *L*(*r*) under CSR. Observed values above the envelope indicate statistically significant clustering at the corresponding distance. (For interpretation of the references to color in this figure legend, the reader is referred to the web version of this article.)

Ripley’s L-function revealed spatial clustering (i.e., AEDs were more grouped than random) in both the 24-h-AED dataset and the all-AED dataset which included devices with restricted access (Fig. 1). At 500-meter scale, the observed L-value for 24-h-AEDs and all-AEDs exceeded the 99 % simulation envelope under CSR, with values of 2,847 (envelope upper bound = 577) and 3,240 (upper bound = 499), respectively. In both cases, complete spatial randomness was rejected, with stronger clustering observed in the all-AED dataset.

Moran’s I values indicated positive spatial autocorrelation (neighboring grid cells tend to have similar AED densities), with *I* = 0.2 for 24-h-AEDs and *I* = 0.4 for all-AEDs. In both cases, the observed statistics were statistically significant (*p* < 0.001), reflecting non-random spatial distributions across the 1 km2 analysis grid.

### Association between AED presence and population or employment density

AED presence was positively and statistically significantly associated with both population and employment density across all four models (Table 1). Standardized effect estimates ranged from *β* = 0.62 [95 % CrI: 0.59–0.66] in the 24-h-AEDs and population model to *β* = 3.25 [95 % CrI: 2.60–3.47] in the all-AEDs and employment model. The corresponding odds ratio per 100 person or employees ranged from 1.10 [95 % CrI: 1.09–1.10] to 1.60 [95 % CrI: 1.46–1.65]. In all models, the posterior mean of the spatial mixing parameter (*Φ*) exceeded 0.997, meaning that nearly all residual variation was spatially structured rather than random.

**Table 1:** Association between AED presence and population or employment density.

Scenario	Standardized *β* (mean) [95 % CrI]	OR_100_ [95 % CrI]	Phi (Φ) [95 % CrI]
24-h-AEDs and Population	0.62 [0.59–0.66]	1.11 [1.10–1.12]	0.997 [0.989–0.999]
All-AEDs and Population	1.58 [1.29–1.75]	1.31 [1.24–1.34]	0.998 [0.992–0.999]
24-h-AEDs and Employment	0.63 [0.58–0.68]	1.10 [1.09–1.10]	0.997 [0.991–0.999]
All-AEDs and Employment	3.25 [2.60–3.47]	1.60 [1.46–1.65]	0.998 [0.992–0.999]

Computed using spatial BYM2 logistic models in 1 km^2^ grid cells. Odds ratios (OR_100_) indicate the change in odds of AED presence associated with a 100-person or 100-employee increase. The spatial mixing parameter (*Φ*) quantifies the proportion of spatially structured variance relative to unstructured heterogeneity. CrI: credible intervals.

### Spatial autocorrelation in AED presence independent of predictors

**Fig. 2:**
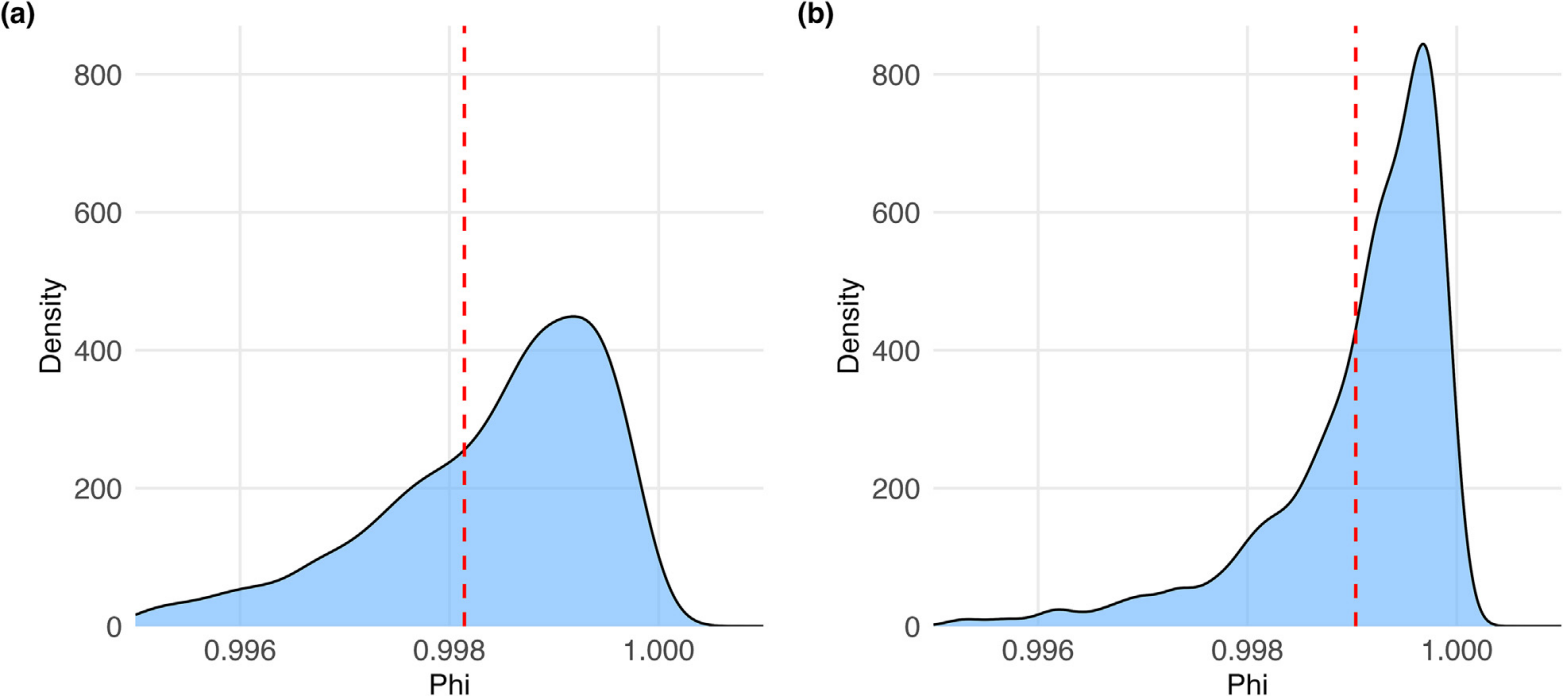
Posterior distributions of the mixing parameter Φ for the intercept-only BYM2 models; (a) 24-h-AEDs. (b) All-AEDs. Phi (Φ) quantifies the proportion of spatially structured variance relative to unstructured heterogeneity. The red dashed lines indicate the posterior mean. (For interpretation of the references to color in this figure legend, the reader is referred to the web version of this article.)

Under intercept-only BYM2 models, the spatial mixing parameter Φ had a posterior mean of 0.998 (95 % CrI: 0.994–0.999) for the 24-h-AED model and 0.999 (95 % CrI: 0.997–0.999) for the all-AED model. Posterior densities were narrowly distributed and peaked near 1.0 in both models (Fig. 2).

### AED accessibility deficit scores

Demographically weighted risk scores varied across scenarios (Table 2). Mean values ranged from 143.2 (all-AED, employment predictor) to 372.6 (24-h-AED, population predictor), with corresponding medians between 20.8 and 65.6. The maximum risk score reached 31,797, observed in the 24-h-AED and employment model. The grid cells falling within the top 5 % of demographically weighted risk scores contained between 16.4 % and 42.0 % of the national population or workforce, with the lowest share in the all-AEDs with employment as a predictor model, and the highest in the 24-h-AEDs with population as a predictor model.

**Table 2:** Summary statistics for demographically weighted accessibility risk scores.

Scenario	Mean risk score	Median risk score	Max risk score	Population in 5 % highest-risk cells	Share of total population (%)	Correlation (*r*)
24-h-AEDs and Population	372.61	65.59	21,623	3,707,877	42.0	0.33
All-AEDs and Population	281.87	60.22	19,645	2,053,253	23.2	0.43
24-h-AEDs and Employment	229.16	28.87	31,797	2,248,276	38.8	0.33
All-AEDs and Employment	143.20	20.83	28,471	952,262	16.4	0.44

Mean, median, and maximum values describe the distribution of demographically weighted risk scores per 1 km^2^ grid cell. The “population in highest-risk cells” refers to the total population or workforce located within the top 5 % of 1 km^2^ grid cells with the highest risk scores. “Correlation (*r*)” reports the Pearson correlation between each cell’s risk score and the mean of its neighbors, indicating the degree of geographic clustering.

For each of the four model scenarios geographic variation in demographically weighted risk scores were visualized through interactive choropleth maps (accessible at https://www.unilu.ch/aedmaps). The maps show spatial heterogeneity in estimated AED under-coverage, with higher risk scores concentrated in specific regions. Pearson correlation coefficients between each grid cell’s risk score and the mean score of its first-order neighbors range from 0.30 to 0.44 across scenarios.

## Discussion

### Summary of key findings

Spatial analysis revealed statistically significant clustering of AEDs. Ripley’s L-function indicated strong deviation from randomness, with clustering more pronounced in the all-AED dataset than in the 24-h subset. Moran’s I further confirmed positive spatial autocorrelation across the 1 km2 grid, again stronger for all AEDs. These results indicate that AED placement is spatially concentrated, especially for restricted-access devices.

AED presence was strongly associated with both population and employment density. Standardized effect estimates ranged from *β* = 0.62 to 3.25, with corresponding odds ratios per 100 persons or employees from 1.10 to 1.60. Associations were stronger with employment density, particularly when all AEDs were included, suggesting that AED deployment aligns more closely with workplace density than residential patterns. In predictor-adjusted models, Φ exceeded 0.997, and in the intercept-only models, Φ reached 0.998 and 0.999, confirming that nearly all unexplained variation followed spatial patterns (i.e., not random noise). This indicates that the AED placement reflects underlying geographic regularities that go beyond metrics of where people live or work.

The top 5 % of grid cells contained up to 42 % of the national population or 16 % of the workforce affected by accessibility deficits. Risk scores clustered geographically (*r* = 0.30–0.44), confirming that AED shortfalls are concentrated in specific zones.

### Implications

AEDs in Switzerland show significant spatial clustering in settled areas rather than random distribution. This suggests deployment inequalities persist even in well-infrastructured, densely populated areas. Clustering was more pronounced in the all-AED dataset, which includes time-restricted devices that are often placed in institutional or commercial settings. International comparisons suggest this pattern is not unique. In Shanghai, AEDs were clustered in transit corridors and government buildings, while surrounding residential areas were underserved.^24^ This pattern introduces both spatial barriers through geographic concentration and temporal barriers through restricted availability, reflecting institutional convenience rather than public health need.

Accessibility is directly tied to patient outcomes.^15^ Studies from other countries have shown that survival rises sharply when shocks are delivered by bystanders with public AEDs, rather than delayed until EMS arrival.^9^, ^10^ In Switzerland, the fact that many devices are locked, restricted or concentrated in workplaces likely means that comparable opportunities for survival are being lost. Experience from São Paulo further illustrates what can be gained when devices are strategically placed and available at all times, with over a third of patients surviving with good neurological function.^11^

These international comparisons align with our regression findings, which reinforce the preference for institutional over residential coverage. AED presence was more strongly associated with employment density than population density, even though 68 % of Swiss OHCAs occur at home, and only 15 % in public spaces.^16^ Similar city-level findings have been reported in Stockholm and Philadelphia.^25^, ^26^ At the national scale, Brown et al. found higher AED prevalence in areas with dense workplaces, however, they did not quantify the independent strength of this association, nor assess potential trade-offs with residential accessibility.^27^ Our results fill this gap, showing that employment density is a stronger predictor than population density and that placement systematically prioritizes institutional visibility over risk. Unlike prior city studies, our national model quantifies this bias across diverse urban and rural contexts and shows that it is embedded in how infrastructure is planned across cantons (Swiss federal states) and not just in major cities.

Even after accounting for population and employment, AED deployment remained highly spatially structured, with Φ exceeding 0.997 across intercept-only models. AED presence is therefore not primarily explained by demographics alone but reflects spatial structuring, where placement follows historical infrastructure or institutional habits rather than current need. Previous studies have linked AED placement with structural features such as transportation infrastructure, commercial areas, nursing homes or elderly population density.^24^, ^26^, ^27^ But most of these studies stop at pattern description and do not quantify the spatial processes underlying deployment decisions. Lin et al. used spatial modeling to identify high-risk areas, yet focused on estimating need rather than explaining persistent deployment choices.^28^ Our analysis isolates this residual structure, suggesting both inefficiency and an absence of strategic oversight. Thus, it is insinuated that without coordinated planning standards, entrenched imbalances persist. Reliance on demographic data alone, without correcting spatial bias, will reproduce inequities. What is needed is a shift from opportunistic placement to oversight mechanisms that prioritize equitable coverage.

Risk model results highlight the scale of placement gaps. The top 5 % of underserved grid cells contained up to 42 % of the national population or 16.4 % of the workforce. These deficits clustered geographically (*r* = 0.30–0.44), confirming their structural nature. Prior studies have examined general misalignments between AED location and OHCA.^25^, ^29^ However, they have not quantified the scale of the population affected, nor have they offered a basis for systematically prioritizing national level high-need zones.

Beyond underserved zones, our analysis also identified areas of overcoverage where AED density exceeded demographic expectation. These clusters, concentrated in economic hubs, mirror the same structural bias, while residential zones remain underserved. Similar redundancy has been documented in city-level studies, but our findings show that it recurs nationally.^24^, ^28^, ^29^ Addressing both deficits and redundancies is essential: without redistribution, expansion risks reinforcing existing biases. Strategic reallocation from overcovered to high-need areas offers a pathway to improve national equity.

Several concrete policy interventions follow from these findings. First, a mandatory national AED register would improve completeness, quality control, and integration with dispatch. Second, ensuring 24/7 accessibility is a low-cost measure to reduce temporal inequities. Third, geospatial risk maps such as those developed here can support systematic prioritization of high-need zones. Finally, relocation offers an efficient complement to expansion, and although we did not model relocation scenarios in this study, future work could quantify the potential gains of such redistribution. A Lausanne study showed that relocating a small share of redundant devices substantially improved coverage at no extra cost,^29^ while work in Fribourg found survival ranged from 8.5 % in urban areas to below 4 % in intermediate rural zones, highlighting persistent regional disparities.^30^ Our findings suggest similar inefficiencies nationwide and the potential to scale relocating strategies across cantons. Embedding such measures in coordinated planning frameworks would shift deployment from opportunism towards equitable, risk-based infrastructure.

### Limitations and strengths

While this study provides a robust national analysis, several limitations should be acknowledged. First, *Defikarte.ch* is a voluntary, community-maintained registry, rather than a mandatory national database. Completeness is uncertain, regionally variable, and may include duplicates or outdated entries; privately purchased devices are included but more likely to be unlisted. Our results therefore represent a conservative lower bound of AED availability. Second, classifying devices into 24-h-AEDs versus all-AEDs simplifies a more nuanced reality of restricted hours, and the all-AED scenario should be read as an upper bound and counterfactual policy experiment. Third, analysis at the 1 km^2^ grid scale assumes uniform population distribution and may overlook cross-border access, which is acceptable for national analysis but may obscure local disparities. Fourth, accessibility was assessed using Euclidean distance, likely overestimating real-world access where terrain or vertical travel causes delays; retrieval times are thus probably longer and coverage gaps even larger than reported. Fifth, population and employment density were used as proxies for OHCA exposure. These make the study replicable in settings without OHCA registries but may misalign with true risk; age adjusted measures would be valuable refinements. Both proxies are also static and do not capture diurnal or weekly shifts: population approximates nighttime and holiday exposure, while employment reflects daytime presence. Using both provides complimentary perspectives, but accessibility metrics remain partial representations of temporal dynamics. Finally, we did not account for points of interest or mobility flows (e.g., transport hubs, tourist sites, schools), so some areas labeled as overcovered under population/employment proxies may in fact reflect intentional, mobility-aligned placement.

The study also has important strengths. It is the first in Switzerland to assess AED deployment nationally while comparing coverage relative to both population and employment density, highlighting structural disparities between residential and institutional access often missed in local studies. The grid-based BYM2 spatial model enhances inference by separating demographic effects from latent spatial structure, thereby quantifying how much of the remaining variation reflects entrenched geographic clustering, rather than demographics alone. The unusually narrow CrIs reflect this strength, arising from complete national coverage with more than 30,000 grid cells and the stabilizing properties of the spatial model, which yield highly precise estimates. Additionally, modeling two availability scenarios (24-h-AEDs and all-AEDs) adds policy relevance by demonstrating how temporal restrictions alter real-world coverage, and by highlighting that spatial placement alone is insufficient if devices are not continuously accessible. Finally, probabilistic predictions, rather than binary classifications, allow more refined risk assessment, identifying both underserved and overcovered zones to support redistribution or expansion based on modeled need.

## Conclusion

AED infrastructure in Switzerland reflects historical placement patterns rather than clinical or population need. Despite national goals to increase bystander defibrillation, spatial modeling reveals persistent inefficiencies not explained by demographics. These patterns highlight structural fragmentation and lack of planning oversight. Improving equity requires moving beyond opportunistic, historically entrenched placement toward strategies anchored in population-weighted accessibility benchmarks and spatial planning tools that systematically target underserved areas.

## CRediT authorship contribution statement

**Sarah Maria Esther Jerjen:** Writing – original draft, Visualization, Validation, Software, Methodology, Investigation, Formal analysis, Conceptualization. **Armin Gemperli:** Writing – review & editing, Validation, Methodology, Conceptualization.

## Funding

This research did not receive any specific grant from funding agencies in the public, commercial, or not-for-profit sectors.

## Data availability

All data used in this study are openly accessible. AED data were obtained from *Defikarte.ch* (https://www.defikarte.ch, accessed December 2024). Population and employment data were provided by the Swiss Federal Statistical Office (https://www.bfs.admin.ch). Settlement data were obtained from the Federal Office for Spatial Development (ARE, https://www.are.admin.ch).

## Declaration of competing interest

The authors declare that they have no known competing financial interests or personal relationships that could have appeared to influence the work reported in this paper.

## Figures and Tables

**Supplementary Fig. A1:**
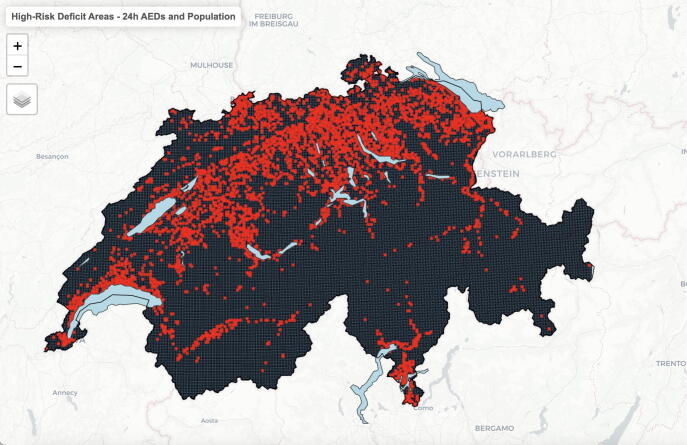
Top 5 % underserved grid cells by population-weighted accessibility deficit, 24-h AED scenario. Static version of the choropleth highlighting the 5 % of 1 km^2^ grid cells with the highest population-weighted accessibility deficits under the 24-h AED scenario. These areas represent zones where the absence of continuously accessible devices affects the largest resident populations.

**Supplementary Fig. A2:**
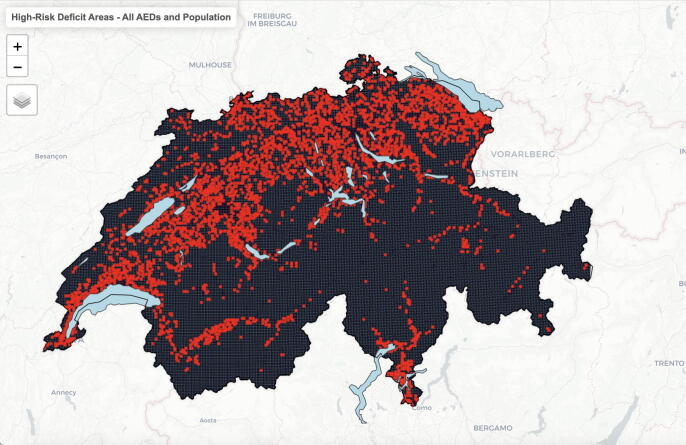
Top 5 % underserved grid cells by population-weighted accessibility deficit, all-AEDs scenario. Static version of the choropleth highlighting the 5 % of 1 km^2^ grid cells with the highest population-weighted accessibility deficits under the all-AEDs scenario, in which all devices are assumed to be continuously accessible. The comparison with illustrates the population-level gains that could be achieved under universal 24-h access.

**Supplementary Fig. A3:**
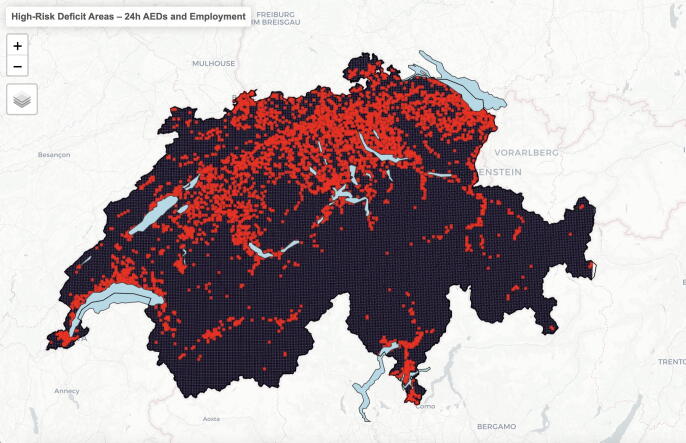
Top 5 % underserved grid cells by employment-weighted accessibility deficit, 24-h AED scenario. Static version of the choropleth highlighting the 5 % of 1 km^2^ grid cells with the highest employment-weighted accessibility deficits under the 24-h AED scenario. These areas represent zones where the absence of accessible devices disproportionately affects large working populations.

**Supplementary Fig. A4:**
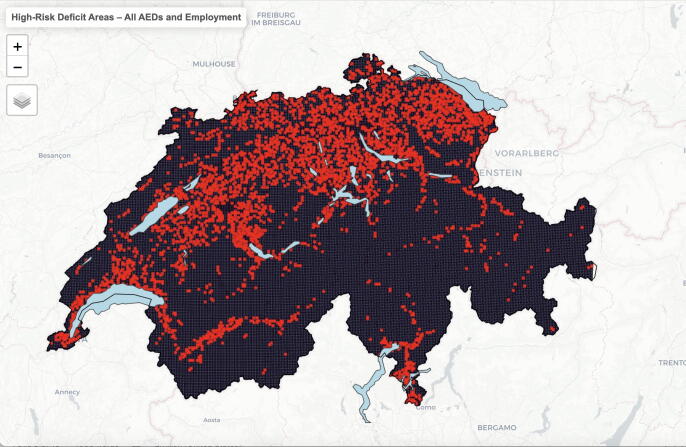
Top 5 % underserved grid cells by employment-weighted accessibility deficit, all-AEDs scenario. Static version of the choropleth highlighting the 5 % of 1 km^2^ grid cells with the highest employment-weighted accessibility deficits under the all-AEDs scenario. These results complement and demonstrate how deficits shift when assuming universal continuous access.
